# Supplementary material for: High Performance Tunable Catalysts Prepared by Using 3D Printing
Source: Materials (Basel). 2021 Sep 2;14(17):5017. doi: 10.3390/ma14175017 (PMC8434323; doi:10.3390/ma14175017)
Supplement: Supplementary file 1 [file materials-14-05017-s001.zip › supplementary.pdf]

materials

Supplementary Materials: High Performance Tunable Catalysts Prepared by Using 3D Printing

Cristian Yesid Chaparro-Garnica [1], Esther Bailón-García [1,2,*], Arantxa Davó-Quiñonero [1], Patrick Da Costa [3], Dolores Lozano-Castelló [1] and Agustín Bueno-López [1]

[1] Department of Inorganic Chemistry, University of Alicante, Carretera de San Vicente del Raspeig s/n, 03080 Alicante, Spain; cristian.chaparro@gcloud.ua.es (C.Y.C.-G.); arantxa.davo@tcd.ie (A.D.-Q.); d.lozano@ua.es (D.L.-C.); agus@ua.es (A.B.-L.)

[2] Carbon Materials Research Group, Department of Inorganic Chemistry, Faculty of Sciences, Campus Fuentenueva s/n, University of Granada, 18071 Granada, Spain

[3] Institut Jean Le Rond d'Alembert, CNRS UMR 7190, 2 Place de la Gare de Ceinture, Sorbonne Université, 78210 Saint Cyr L'Ecole, France; patrick.da_costa@sorbonne-universite.fr

* Correspondence: estherbg@ugr.es

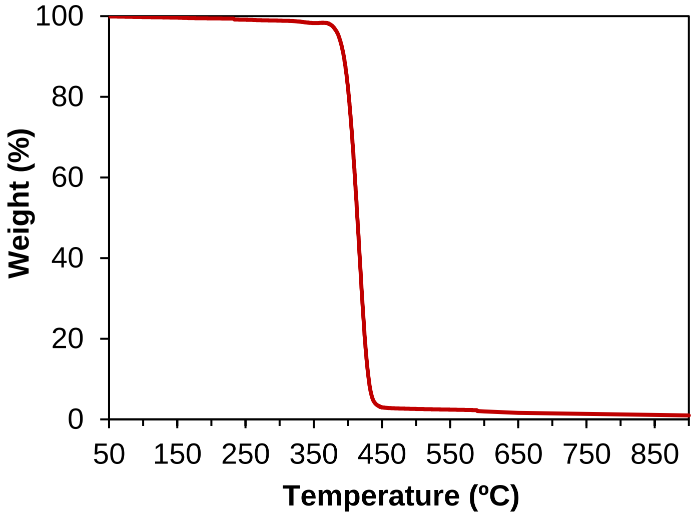

Figure S1. Thermogravimetric analysis of CPE+ template under N_2 atmosphere.

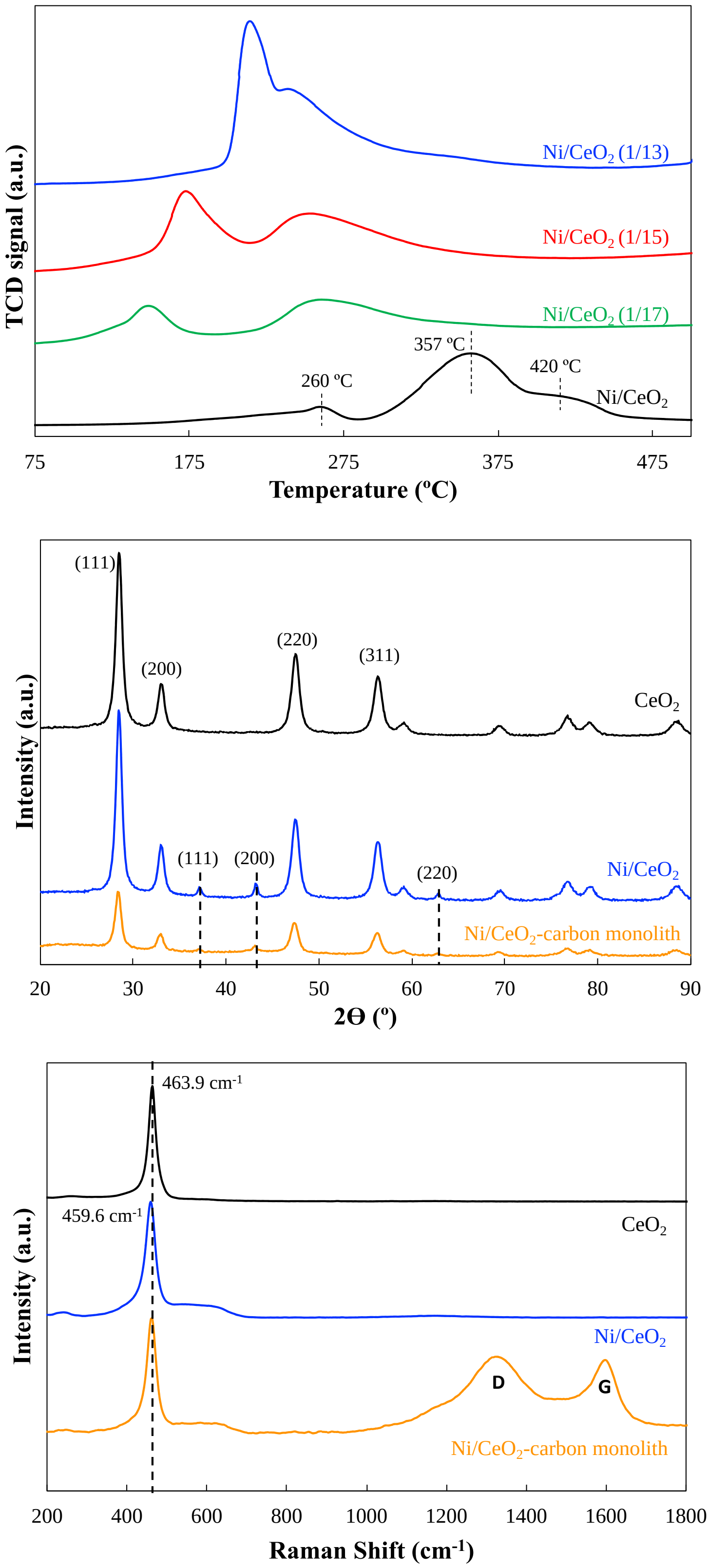

Figure S2. A) H_2-TPR profiles and B) XRD and C) Raman patterns of CeO_2 and Ni/CeO_2 catalysts, both powdered and supported (Ni/CeO_2-carbon monolith).

© 2020 by the authors. Submitted for possible open access publication under the terms and conditions of the Creative Commons Attribution (CC BY) license (http://creativecommons.org/licenses/by/4.0/).
